# Implementing a health labour market analysis to address health workforce gaps in a rural region of India

**DOI:** 10.1186/s12960-022-00749-6

**Published:** 2022-06-04

**Authors:** Samir Garg, Narayan Tripathi, Michelle McIsaac, Pascal Zurn, Tomas Zapata, Dilip S. Mairembam, Niharika Barik Singh, Hilde de Graeve

**Affiliations:** 1 State Health Resource Centre, Raipur, Chhattisgarh India; 2 Health Workforce Department, WHO, Geneva, Switzerland; 3 WHO, South East Asia Regional Office, New Delhi, India; 4 WHO, India Office, New Delhi, India; 5 Government of Chhattisgarh, Raipur, India

**Keywords:** Health Labor Market Analysis, Health workforce, Human Resources for Health, Underserved areas, Rural areas, Supply and demand, India, WHO, Workforce strategy, Workforce 2030

## Abstract

**Background:**

Human Resources for Health (HRH) are essential for making meaningful progress towards universal health coverage (UHC), but health systems in most of the developing countries continue to suffer from serious gaps in health workforce. The Global Strategy on Human Resources for Health—Workforce 2030, adopted in 2016, includes Health Labor Market Analysis (HLMA) as a tool for evidence based health workforce improvements. HLMA offers certain advantages over the traditional approach of workforce planning. In 2018, WHO supported a HLMA exercise in Chhattisgarh, one of the predominantly rural states of India.

**Methods:**

The HLMA included a stakeholder consultation for identifying policy questions relevant to the context. The HLMA focused on state HRH at district-level and below. Mixed methods were used for data collection and analysis. Detailed district-wise data on HRH availability were collected from state’s health department. Data were also collected on policies implemented on HRH during the 3 year period after the start of HLMA and changes in health workforce.

**Results:**

The state had increased the production of doctors but vacancies persisted until 2018. The availability of doctors and other qualified health workers was uneven with severe shortages of private as well as public HRH in rural areas. In case of nurses, there was a substantial production of nurses, particularly from private schools, however there was a lack of trusted accreditation mechanism and vacancies in public sector persisted alongside unemployment among nurses. Based on the HLMA, pragmatic recommendations were decided and followed up. Over the past 3 years since the HLMA began an additional 4547 health workers including 1141 doctors have been absorbed by the public sector. The vacancies in most of the clinical cadres were brought below 20%.

**Conclusion:**

The HLMA played an important role in identifying the key HRH gaps and clarifying the underlying issues. The HLMA and the pursuant recommendations were instrumental in development and implementation of appropriate policies to improve rural HRH in Chhattisgarh. This demonstrates important progress on key 2030 Global Strategy milestones of reducing inequalities in access to health workers and improving financing, retention and training of HRH.

**Supplementary Information:**

The online version contains supplementary material available at 10.1186/s12960-022-00749-6.

## Background

No health system can perform effectively without adequate Human Resources for Health (HRH) [1]. The target 3-c of the Sustainable Development Goals specifically calls for—“substantially increase health financing, and the recruitment, development and training and retention of the health workforce in developing countries” [2]. While the importance of HRH is getting better recognized now, health systems in most of the low- and low- to middle-income countries (LLMICs) continue to suffer from serious gaps in health workforce [3–8].

Health Labor Market Analysis (HLMA) has been used to identify and address workforce challenges in many countries over the last decade [4, 9–13]. HLMA can help countries to identify the gaps in their HRH policies which affects the dynamics of labour market including policies related to wages and retention, training, geographic distribution, skill mix, unemployment and gender inequities. This can enhance understanding of the factors that constrain HRH and result in more effective policies and interventions to address these [4, 14].

The Global Strategy on Human Resources for Health—Workforce 2030 was adopted in the World Health Assembly in 2016 [15]. The strategy prominently includes HLMA as a necessary input for building evidence based HRH agenda and improving health workforce issues and policies across the globe. WHO has also committed itself to providing normative guidance and technical support to countries and states on HLMA and streamlining their national and state HRH strategies [15].

Like many other LLMICs, India is also facing the severe HRH challenges [6, 16–21]. India has an overall shortage of HRH in relation to the size of its population and rising epidemiological needs [16, 18]. One fundamental issue in India is that availability of national data on HRH has been poor. Particularly, the registration data and facility based HRH data are incomplete and unreliable [17, 22, 23]. This suggests that India still has progress to make on the Global Strategy milestone of health workforce registries to track health workforce stock, distribution, flows, demand, supply, capacity and remuneration. Most of the existing studies in India have relied on employment data from the national sample surveys or national decadal census. These studies have focused mainly on stock and composition of HRH which is mostly related to the supply side of the health labour market [16–19, 22]. As a result, many policy levers for addressing HRH have received inadequate attention in India. Thus HLMA based studies on HRH in India are much needed.

Chhattisgarh is one of the poorest states in India [24]. It has a population of 29 million which is predominantly rural and tribal [25, 26]. Formed in year 2000, Chhattisgarh inherited a health system with severe deficiencies in HRH capacity [27]. The state has a mixed health system. The private hospitals accounted for around 40% of inpatient care episodes in 2019 [25]. In case of outpatient care, the share of formal and informal private providers was 23% and 38%, respectively [28]. The public sector facilities are organized in different tiers—a Sub Health Centre (SHC) at 3000 to 5000 population, a Primary Health Centre (PHC) at 20 000 to 30 000 population, a Community health Centre at 80 000 to 120 000 population and a District Hospital (DH) at around a million population [28]. DH and CHC provide secondary and primary care, whereas PHCs and SHCs focus exclusively on primary care [28].

The state was a pioneer in starting a statewide programme of CHWs [28–30]. Another significant innovation of the state in HRH field was of non-physician clinicians known as Rural Medical Assistants (RMAs) to provide primary care [27].

In 2018, Chhattisgarh state requested WHO to provide technical support for addressing the HRH gaps. Given the advantages HLMA offers in clarifying HRH issues, WHO supported a HLMA in Chhattisgarh. The HLMA was initiated in September 2018 in collaboration with State Health Resource Centre (SHRC), a technical organization providing support to the state [23]. The current study presents the results of the above HLMA carried out in Chhattisgarh state and aims to find out the extent to which the recommendations of HLMA were implemented in the state and ultimately if improvements were achieved with respect to HRH in the state.

## Methods

### HLMA framework

**Fig. 1:**
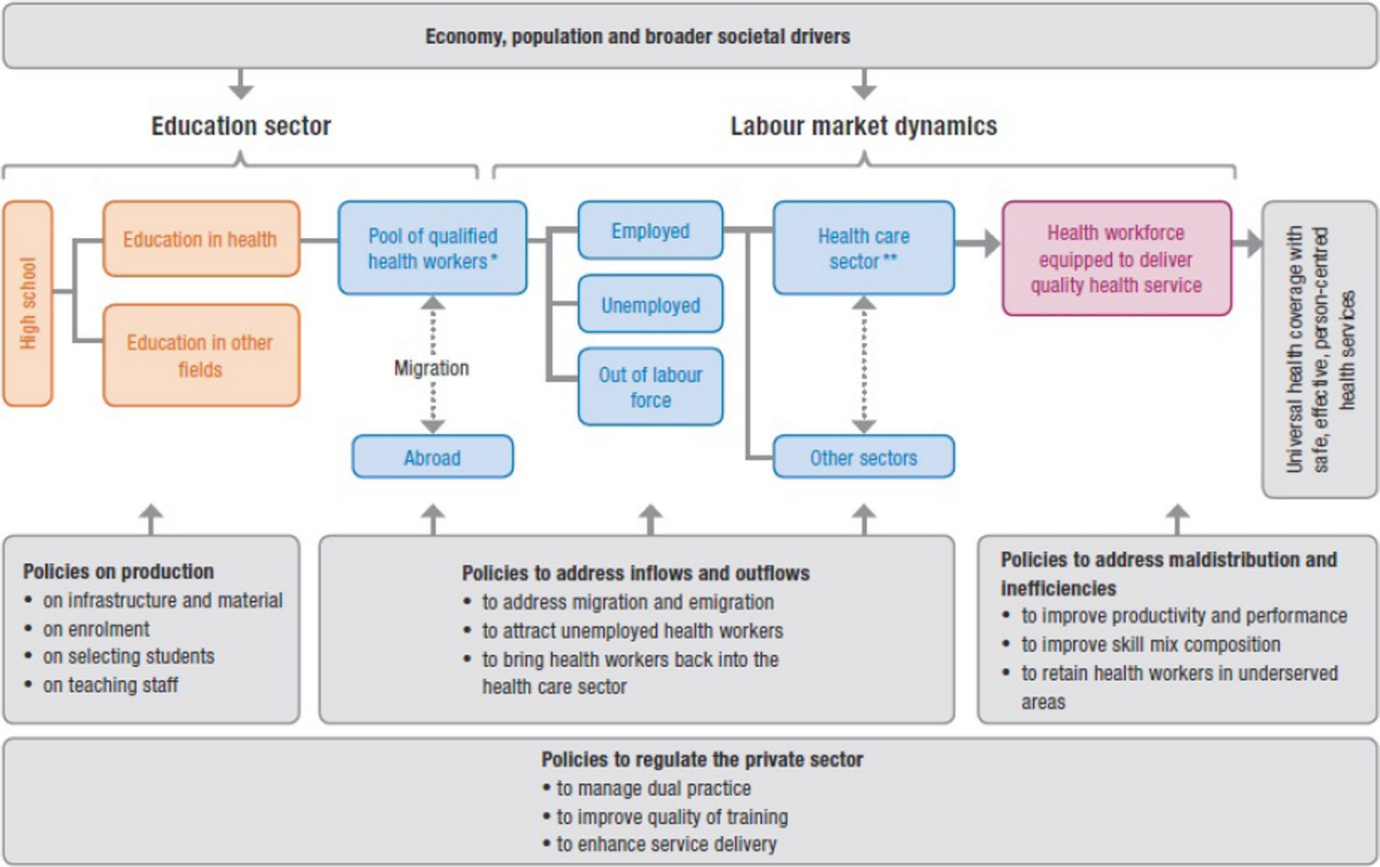
HLMA framework for UHC [14]

The study applied the HLMA framework as proposed by Sousa et al., reproduced here as Fig. 1 [14]. According to this framework, the health labour market is a dynamic system comprising two distinct but closely related elements, that is, the supply of health workers and the demand for such workers. The above framework also includes the generic policy levers for addressing the issues in health labour market.

### Data collection and analysis

The study used a comprehensive multi-pronged approach to data collection and analyses. Table 1 describes the kind of data collected for the required purposes at various stages of the HLMA. It also describes the methods used to analyse the data.

**Table 1:** Data collection and analysis for different stages of HLMA

Sl.	Purpose	Data collection methods	Data analysis
a	Identification of key policy questions for HLMA in Chhattisgarh	Stakeholder consultation was carried out in 2018 to identify the key policy questions. Qualitative semi-structured interviews were conducted with key informants of relevant stakeholder categories that included—state level senior leadership of health department, state officials directly involved in implementing HRH policies, district and sub-district-level health officials, officials from teaching hospitals and training institutions, health professionals, health experts from the civil society in Chhattisgarh and representatives from private hospitals. The detailed list of stakeholders interviewed is given in Additional file 1: Table S1. The consultations were facilitated by technical experts from the WHO	Thematic analysis was done of data collected through interviews
b	Understanding the existing literature	Web search was conducted on the pubmed and google scholar. The key words were decided to search the existing studies on HRH gaps, strategies and policies on health workforce issues using key words in Chhattisgarh state. The international literature on HLMA was also searched using the above mentioned procedure	Desk review of the relevant studies was done
c	Understanding existing HRH policies, rules and procedures in Chhattisgarh	Government documents on HRH including rules and procedures related to recruitment, policies for retention and incentives for HRH, training, promotion and transfers and budget documents were collected from concerned sections of the Department of Health in Chhattisgarh. The relevant documents were identified by seeking information from the key informants directly involved in implementing HRH policies at state level	Desk review of the relevant documents was done
d	Assessing production, absorption, recruitment and geographic distribution of HRH in Chhattisgarh	Secondary quantitative data were collected from various sections of the Department of Health. This was the main source of data for the quantitative analysis. It included data on the district-wise number of approved and filled positions of different healthcare professionals, recruitment drives, numbers of health professionals registered under professional regulation bodies and numbers of health professionals produced by training institutions. The detailed list of data collected along with period and source is given in Additional file 1: Table S2	Data were entered or imported in Microsoft excel and presented in frequencies and percentages. It was analysed for different dimensions, e.g. production and recruitment; geographic distribution and vacancies
e	Identifying underlying reasons for HRH gaps	In-depth interviews of key informants among different stakeholders were conducted. Interview guides were prepared for the different categories of stakeholders and those were focused on specific gap relevant to them. The detailed list of stakeholders interviewed for this purpose is given in Additional file 1: Table S1	All interview recordings were transcribed in digital files and each transcript was read carefully. Transcript was annotated using different labels and codes. Data were conceptualized creating themes and grouped in thematic categories and sub-categories
f	Assessing changes in HRH situation from September 2018 to August 2021, i.e., 3 years after initiating HLMA	Data were collected on filled positions of various cadres till 2021. The data points on which information was collected up to 2021 are indicated in Additional file 1: Table S2	Data were analysed quantitatively in Microsoft excel. Comparison tables were developed to measure the changes in HRH vacancies
g	Assessing policy changes in HRH 3 years after initiating HLMA	For each recommendation in HLMA, information was sought on policy changes from key informants directly involved in implementing HRH policies. Relevant documents were collected	Desk review of the relevant documents was done

## Results

The results have been presented in the following structure:

- Policy questions for HLMA.
- Analysis of the existing density, production, absorption and geographical distribution of under-graduate (UG) doctors and relevant qualitative findings.
- Findings on specialist doctors, nurses and Community Health Officers (CHOs).
- Recommendations from HLMA.
- Changes in HRH policies and workforce vacancies after 3 years of initiating HLMA.

### Stakeholder consultation

Stakeholders expressed the need to focus the HLMA on availability and distribution of HRH in public sector. There was a consensus that there was a shortage of HRH under government employment in clinical roles, with a large number of vacancies of doctors and nurses in government facilities. Based on the above gaps identified by the stakeholders, the following policy questions were considered for the HLMA in Chhattisgarh:

- What factors are driving the shortage of clinical cadres in public sector—medical specialists, doctors, nurses and Community Health Officers (CHOs)? Is their production in Chhattisgarh sufficient to meet their current demand?
- How to improve the recruitment and deployment of the health workforce in Chhattisgarh, especially for rural and remote areas?

## Doctors

### No. of doctors working in the state

The density of doctors in Chhattisgarh was 2.9 per 10 000 population as compared to the national average of 7.6 qualified doctors per 10 000 population in 2018 [18]. The state did not have any significant in-migration of doctors from other states. In addition around 300 doctors were exiting the HLM annually due to retirement or death [23]. Chhattisgarh had not decided any specific norm for the desired density of doctors [23]. If the state were to aim to reach the national average, it would need a total of around 22 900 doctors for the current population of 29 million in 2018. This translates into adding 14 600 more doctors to its current strength of around 8300 doctors in 2018.

### Production of doctors

At the time of formation of the state, there was only one teaching hospital in the state and it produced 150 doctors annually. By 2018, there were seven public and two private teaching institutions in the state with combined capacity to produce 750 doctors annually. The number of doctors graduating annually in the state was 2.59 per 100 000 population. This was around half in comparison to the Indian average (5.2 per 100 000) [22]. If the state were to reach the national average, it will need to double the production of doctors, i.e. from 750 to 1500 annually. In a decade thus, 15 000 more doctors can be added, while the population reaches around 32.5 million. Taking into account the expected retirements over a decade, the state can have around 22 500 doctors. This would translate into a density of 7 doctors per 10 000, which is close to the current national density of 7.9 per 10 000.

The existing medical colleges in the state, both government and private colleges however are facing challenges in getting adequate number of faculty to run the MBBS programme. Analysis of data collected from teaching institutions shows that around 40% of the faculty posts were vacant in government medical colleges in 2018. As a result, an increase in intake in MBBS could be a feasible option only if it can be combined with an increase of faculty.

### Distribution of doctors

Around 46% of the doctors in the state were employed in public sector. This is higher than the national average where around 35% of the existing doctors are estimated to be working in public sector in 2018 [18].

**Fig. 2:**
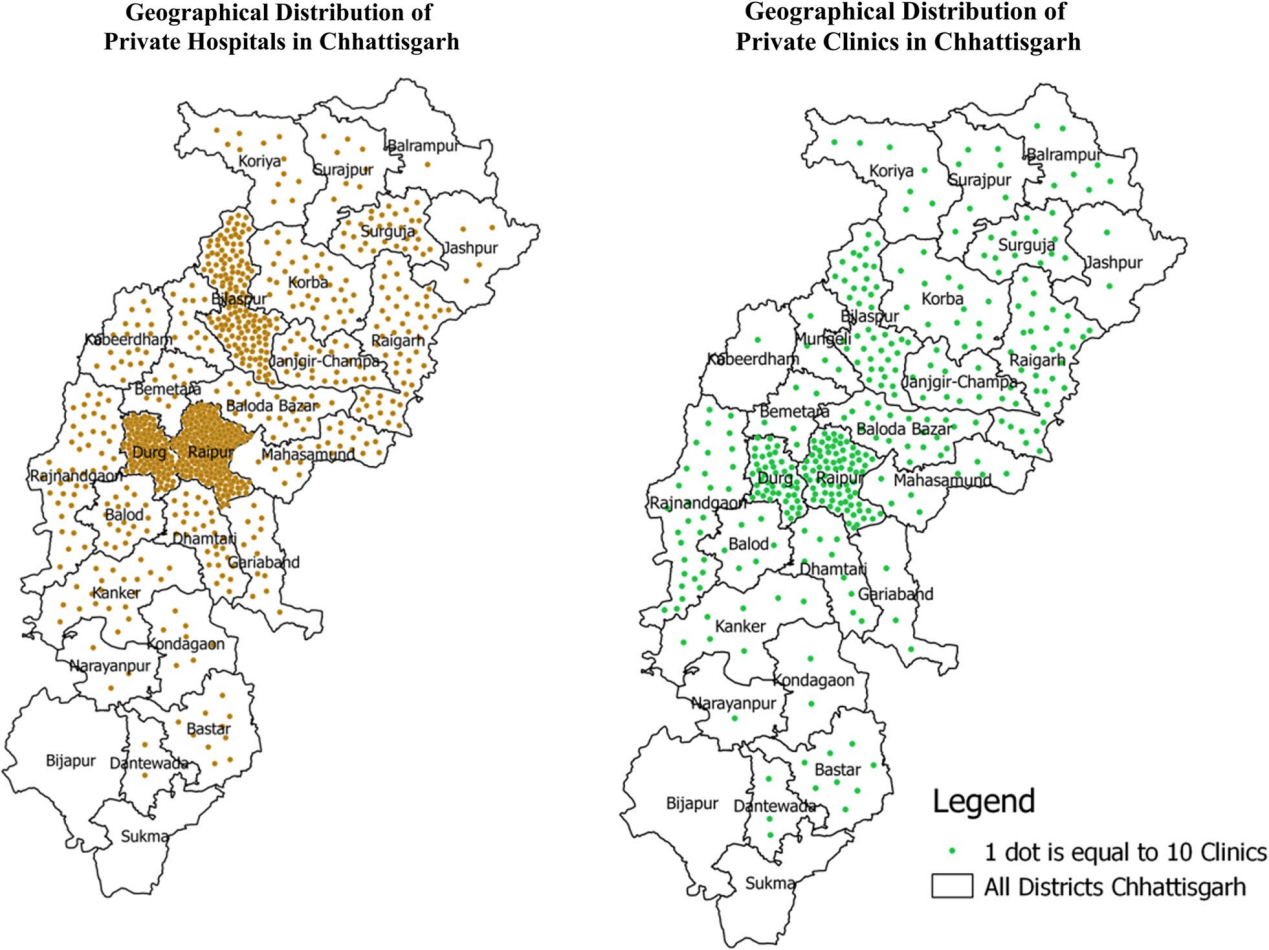
Geographical distribution of private hospitals and clinics in Chhattisgarh, 2018 (Source: Authors’ depiction of data collected from Directorate of Health Services, Chhattisgarh)

There were 955 private hospitals and 3614 private clinics registered in the state in 2018. As the maps in Fig. 2 show, the private facilities were concentrated in central plain regions where the main urban centres of the state are situated.

Healthcare in rural areas, i.e. most district headquarters and below was dependent on services run by government. The public sector however suffered from shortages of doctors. Out of the 2456 positions sanctioned for under-graduate (UG) doctors for public facilities at district headquarters and below, 43% were vacant in 2018.

### Absorption of UG doctors

The state showed poor absorption of UG doctors it had produced. In the 18 year period since its formation, the state was able to recruit around 20% of the total 3500 doctors produced in the state in the above period. Table 2 provides the number of doctors recruited in each drive.

**Table 2:** Recruitment drives conducted for UG doctors in Chhattisgarh—year 2000 to 2018. Source: Data collected from Directorate of Health Services, Chhattisgarh

Year of recruitment drive	No. of UG doctors recruited
2000	91
2005	77
2009	21
2011	125
2014	128
2016	214
Total	656

No recruitments took place in 2017 and 2018. While the vacancies of doctors were high overall, there were regional variations in the vacancies of doctors. The situation was worse in rural and tribal areas. The highest rate of vacancies of UG doctors was in the predominantly tribal region of Bastar (61%) whereas the central and more urbanized regions of Raipur and Durg had the least vacancies (31%).

### Policies to attract under graduate doctors

In order to attract doctors to remote areas, the state had implemented a policy in 2011 to give cash incentives for doctors and other health workers working in rural and remote areas [31–33]. The amount of incentive however did not increase over the years. The incentive for a doctor was around 30% above the salary in 2018. Qualitative analysis of interviews suggested that in 2018 this amount was no longer perceived as attractive enough for UG doctors to work in rural and remote districts.

The state had a bond-scheme for freshly graduating UG doctor to work in rural areas. A bond meant that each UG doctor graduating in the state had to mandatorily work for at least 2 years with government. Another policy to attract UG doctors to work in remote areas was to give them additional marks in the entrance examination for post-graduate courses. Qualitative analysis indicated that the above policies were effective in attracting UG doctors to join under annual contracts.

## Specialist doctors

The gaps in absorption and distribution were even more severe in case of the Specialist doctors, i.e. those with post-graduate qualifications in clinical streams. Of the 8300 doctors working in the state in 2018, 3320, i.e. around 40% were specialist doctors.

The teaching institutions of the state had a capacity to train 230 post-graduate doctors annually of which 100 seats were introduced in 2018. The capacity in 2018 represented 0.9 specialists trained annually per 100 000 population. The corresponding rate for India was 3.1 specialists per 100 000 population [23]. Sri Lanka had a similar rate as Chhattisgarh with 0.93 post-graduate doctors produced per 100 000 population [10]. The state was a net recipient of specialists from other parts of the country, but most of them joined the private sector or the government teaching hospitals in cities. According to the data collected in HLMA, around 49% of the 3320 specialists in the state in 2018 were employed in private sector.

For the district level and below, there were 1726 sanctioned posts in government of which 88% were vacant in 2018. It was further alarming that the absolute number of specialists in regular employment was declining. In the 3-year period preceding 2018, there was a decline of 18%. No recruitments of specialists took place at district or below since the state’s formation in year 2000. The age profile of above specialists collected during the HLMA showed that and a majority of them were close to retirement in 2018.

Analysis of government rules showed that the state did not have an entry cadre for Specialists for facilities in district headquarters and below. A post-graduate doctor had to join at the same rank and salary as an UG doctor. Qualitative analysis showed that this was a key barrier to recruitment of Specialists. After 5 years of service, a post-graduate doctor became eligible for a promotion to a specialist position. It was found that there were long delays in promotions due to administrative gaps. In 2018, there were around 300 doctors with post-graduate qualifications who were awaiting their promotion to specialist cadre. Being posted as general physicians, many of the specialists lost their specialized skills. The specialists in such role resented being assigned to the general and emergency care duties similar to UG doctors.

Another barrier in attracting specialists was the inadequate remuneration. Salaries of specialists in government service did not keep pace with salaries in private sector. A specialist working in public sector on an average earned around 45% less salary than a counterpart employed in private sector. The vacancies of specialists in remote and rural districts were as high as 95%. Bijapur, one of the remotest districts had shown the way by successfully hiring specialists from neighbouring states by offering them remuneration similar to the rates prevalent in private sector [32, 33].

Qualitative interviews with freshly passed post-graduate doctors revealed a perception that the place of posting for new entrants in government jobs were not allocated through a transparent process. According to the other stakeholders interviewed, the state did not have a transparent transfer policy.

The state had implemented a compulsory service bond for post-graduate doctors from 2000 onwards [31]. There were around 100 post-graduate doctors working under the bond-scheme in 2018, but most of them worked in government-run teaching hospitals in urban centres.

### Policies for task sharing

Keeping the chronic shortage of Specialists in mind, Chhattisgarh had pioneered a policy to promote task sharing. It introduced multi-skilling of UG doctors in obstetric care and anaesthesia skills to cover the gap in capacity to provide emergency obstetric care in rural public facilities in 2003 [34]. The initiative became part of a national policy. According to the qualitative interviews of stakeholders, the results of the above multi-skilling initiative in achieving actual task sharing were found to be mixed. Though the policy was able to help in expanding the obstetric care services to some extent, multi-skilling in other specialist skills had not been attempted.

## Nurses

The main cadre of nurses in the state is known as Staff Nurses (SN). They have around 4 years of education in nursing and they are posted in facilities with inpatient services. There were around 17 000 SNs working in Chhattisgarh in 2018. Around 53% of them were working in private sector and rest in government facilities [10].

At district hospital and below, 3823 SNs were working against 6502 sanctioned government positions. It represented a vacancy rate of 39%. The vacancies were greater in remote areas. For example, the remote division of Bastar had 58% posts vacant, whereas the vacancies were 32% in the centrally located and urban division of Raipur.

In 2018, 87% of the nursing colleges in the state were in private sector. They had a combined capacity to produce 6635 nurses annually. Qualitative interviews with stakeholders showed that the quality of education provided in many of the private schools was poor resulting in production of nurses with inadequate skills. There were around 25 000 qualified nurses residing in Chhattisgarh in 2018 and around 8000 of them were unemployed. This indicated that there was a situation of supply exceeding the demand for nurses due to over-production.

Yet, there were vacancies in government facilities. It was found that the salaries of nurses employed under annual contracts were 45% lower than those employed permanently as regular cadre. Yet, 74% of contractual posts were filled as opposed to 57% of the permanent positions. For the permanent posts, it was mandatory to seek permission from the state finance department before recruiting. Filling the regular, i.e. permanent positions required considerable administrative capacity. The advertisements for the positions attracted a large number of applicants and an examination had to be conducted to make the selection possible. Health department had not developed its capacity to conduct free and fair examinations. It depended on another specialized agency set up by government for conducting examinations for the purpose of selection. This dependence often resulted in long delays in completing the examination process.

## Community health officers (CHOs)

This cadre is meant to lead the provision of primary care services through the Health and Wellness Centres (HWCs). In order to become CHOs, nurse graduates undergo additional training of 6 months. The training was conducted at district hospitals and certification was provided by India’s national Open University [35]. The state started the above course in 2018. The state planned to establish 4414 HWCs by year 2022 and therefore needed to produce an equal number of CHOs by then. In the first batch of training, 70 CHOs completed the training in 2018. The state established a capacity in 2018 to train 960 CHOs annually. The key concern beyond the production of CHOs was regarding their regional distribution. Without an enabling policy the remote districts could get deprived in getting the required number of CHOs.

## Actions recommended by HLMA and their implementation

Based on the above analysis, a set of key actions were recommended to the state government, which were followed up through SHRC. Table 3 lists the recommendations and the status of their implementation till August 2021.

**Table 3:** Recommendations of HLMA in Chhattisgarh and the status of their implementation

Cadre	Recommendation	Change—from September 2018 to August 2021
Specialist doctors	Organize drives to complete the backlog of promotions of PG doctors who have already served 5 years or more	The recommendation was implemented, resulting in addition of around 230 specialists
	Change rules for recruitment of Specialists—to allow direct recruitment of post-graduate doctors into Specialist cadre	The policy change came into effect in June 2021. Its success in attracting specialists is yet to be studied
	Provide a one-time relaxation for promoting existing post-graduate doctors with less than 5 years service	Not implemented
	Increase salaries including through use of flexibility available for hiring on annual contracts	Districts used their flexible resources to attract PG doctors, resulting in recruitment of around 100 more specialists
	Increase the amount of incentive for working in remote areas	A modest increase was proposed by the state government but it could not secure the approval of central government who funds this incentive
	Transfer policy to help tribal areas: introduce a policy for each doctor to work for a mandatory fixed term (5–7 years) in rural and remote areas	Not implemented
	Multi-skilling of UG doctors by training them in specialist skills through short courses	Initiated
UG Doctors	Organize recruitment drives frequently	Implemented. At least one drive took place annually
	Transfer policy to help tribal areas	Not implemented
	Transparent allocation of place of posting after recruitment	Implemented
Nurses	Organize recruitment drives	Implemented
	Quality assurance in private nursing schools	Implemented partially
CHOs	Increase training capacity and quality	Implemented
	Organize recruitment drives	Implemented but faced challenges due to litigations
	Regional quotas to ensure that remote/underdeveloped districts also get CHOs	Implemented
	Continued capacity building—in-service training	Initiated

**Fig. 3:**
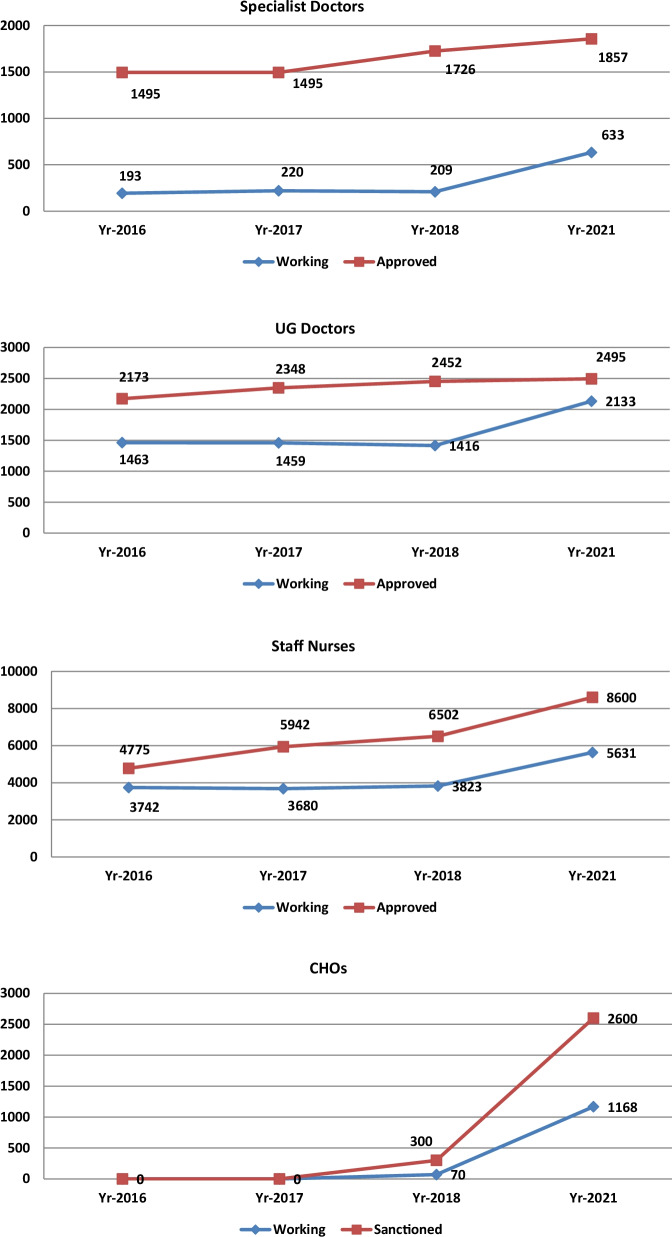
Gap between approved posts and working post of key cadres in Chhattisgarh (Source: authors’ depiction of data collected from different directorates under department of health [detailed list of data is available in Additional file 1: Table S2])

Figure 3 shows the changes in availability of the key clinical workforce at district level and below.

Table 4 provides a summary of key changes achieved in HRH along with the remaining gaps.

**Table 4:** Changes in key HRH in Chhattisgarh’s public facilities from September 2018 to August 2021, current gaps and potential solutions

Cadre	Changes in HRH in public facilities	Current gaps and potential strategies
Specialists	• The number of specialist doctors increased by 203% with addition of 424 specialists • The above increase was due to two main reasons: (a) there was progress in completing the due promotions (b) flexible salaries were implemented by districts	• Despite the impressive increase in number of specialists, a considerable gap remained with 66% vacancies • The recent change in policy allowing direct entry of PG doctors in specialist cadre may help in attracting more specialists in future • The success in improving availability of specialists so far has been largely limited to district hospital level. The approach of using flexible salaries to attract specialists can be extended to get more specialists at CHC level also. The short training courses for UG doctors can also help in task sharing. Funds were secured for a PG diploma course in family medicine and its implementation can help in multi-skilling of UG doctors
UG doctors	• The number of UG doctors increased by 51% with addition of 717 doctors. It brought down the vacancies dramatically from 43 to 15%. This could be achieved by the department by increasing its management capacity to handle large recruitment drives	• Continuous skill building of recruited doctors will be needed
Staff Nurses	The number of Staff Nurses increased by 47% with addition of 1808 nurses. The quality assurance drive resulted in around one-fourth of the private schools being asked to improve quality standards. Eventually, 13 schools (around 10% of total) who did not improve were barred from taking new admissions	• Continuous skill building of recruited nurses will be needed • Further recruitment drives are needed for filling the new contractual posts
CHOs:	The number of CHOs increased manifold with addition of 1098 CHOs. Implementing the regional quotas helped some of the remote districts. In-service training was initiated for CHOs joining the HWCs and most of them were trained by SHRC on standard treatment protocols for primary healthcare services. The production capacity was increased to 1600 CHOs per year. Medical Colleges have been roped in to enhance quality of training	• The state lost opportunity to train another 1400 CHOs by 2021 due to litigations. Alternative strategies need to be found for quickly recruiting CHOs in large numbers

## Discussion

The HLMA in Chhattisgarh found that public sector services played a central role in providing access to healthcare needed by the rural population while the formal private providers were largely concentrated in urban centres. Earlier studies in Indian context have also reported such a pattern [28, 30]. A significant part of healthcare utilization was from the informal or unqualified private providers and the quality of such care has been poor [17, 28]. This highlights the importance of examining the distribution of key HRH cadres across public and private sectors and their role in coverage of rural population. Recommending a blanket number of doctors per unit population may not help in improving access for those who need it the most.

The current study found that there was a widespread perception that WHO has recommended 1 doctor per 1000 population for all countries in the world. Though WHO has never made this recommendation; media reports, academic studies and policy documents in India continue to propagate this perception [36, 37]. This erroneous perception has often resulted in advocacy to increase the production of doctors in India [36, 37]. The HLMA showed that the production of health workers was not the central issue in case of Chhattisgarh. The poor absorption by the public sector emerged as the main problem, particularly for doctors and nurses. Increasing the production of HRH alone may not help if the public sector does not absorb this additional capacity. HLMA studies in other LLMICs have also played an important role in clarifying this aspect [4, 11]. Studies have shown that comprehensive policies informed by HLMA are needed to address health workforce issues and just addressing the supply side, i.e. increasing the production may not help [4, 11, 13, 38, 39].

Regarding absorption capacity, it was found that while attracting doctors through better remuneration could help the remote areas; the key was in fact more technical notably, the organization of frequent and regular recruitment drives. As such, there were capacity gaps and governance issues that created challenges in regularly organizing recruitments. A key barrier was the requirement of seeking mandatory approval from the finance ministry each time any recruitment was to be done. This indicates the need for governments to streamline processes. As suggested by the Global Strategy an institutional mechanism to coordinate the intersectoral health workforce agenda is needed to facilitate addressing this requirement for recruitments under health ministry given that healthcare is an essential service and a human right.

The study illustrates that in India, the public sector can quickly fill HRH vacancies, including in rural areas. Technical barriers were also found in the case of specialist doctors. The importance of multi-skilling based strategies to allow task sharing so that UG doctors can handle many of the secondary care functions may be a more impactful solution. This in turn would require attention to building clinical skills in doctors through in-service training [40]. A study on HLMA in Timor Leste has also highlighted the importance of ensuring training and mentoring throughout the career for health workforce [41].

The study found a situation of excessive production of certain health workers, particularly through private sector education. For example, nurses where ensuring the quality of education is a central issue. The importance of regulating quality of education in private sector was demonstrated, as without this the health labour market may not absorb them due to uncertainty regarding their skills. A similar concern has been raised by other HLMA based studies in LLMICs [4]. Addressing these concerns, notably the oversight of private sector and accreditation mechanisms, form part of the global milestones for the Global Strategy 2030.

The issues which could not be resolved were those involving a substantial increase in government expenditure. HLMA studies in Peru and Kenya had also recommended an increase in salaries to attract and retain doctors [11, 42]. This indicates the possibility that poor salaries may be a constraining factor on the demand side in attracting doctors in many LLMICs. This may, as suggested by the Global Strategy, indicate a role for the international community to strengthen sustainable support for HRH, including the possibility for investment in capital and recurrent expenditure (including salaries).

The current study could not fully address issues like the dual practice, productivity and performance of the deployed HRH, gender dynamics and career pathways. Future research and HLMA should aim to address these key areas as existing studies have shown the importance of many of the above issues in LLMIC contexts [4]. Retention of doctors was not a focus of the current study, as separate studies were supported by the WHO to cover this aspect [32, 33].

## Conclusions

The HLMA revealed large vacancy rates for health workers in Chhattisgarh which were trending upwards across time. While the most urbanized areas had almost no vacancies, the more rural, remote and tribal areas of the state had extremely high vacancy rates. Paradoxically, the increase in vacancy rate had occurred against a backdrop of rising numbers of trained qualified health workers. Pragmatic policy recommendations were developed to strengthening the effectiveness of recruitment, facilitate promotion, and transfer policies, improve medical skills and quality standards and implement time-bound recruitment plans for medical officers and nurses.

The HLMA and the pursuant recommendations were instrumental for the state to mobilize resources and establish new programmes for in-service capacity building. Over the past 3 years since the HLMA began an additional 4547 health workers including 1141 doctors have been absorbed by the public sector. The number of specialist doctors increased by 203%, UG doctors by 51% and nurses by 47%. The vacancies in most of the clinical cadres were brought below 20%. This demonstrates important progress on three key 2030 Global Strategy milestones. Firstly, this demonstrates important progress in reducing inequalities in access to health workers across the state. Secondly, it demonstrates the effort to create, fill and sustain additional full-time jobs in health and social care sectors to address the needs of underserved population. Thirdly, important steps were taken by the state to increase health financing for HRH and recruitment, development, training and retention. All of which brings Chhattisgarh a step closer to achieving its UHC goal.

## Supplementary Information

**Additional file 1: Table S1.** List of stakeholders interviewed (qualitative). **Table S2.** List of quantitative data collected in Chhattisgarh.

## Data Availability

The datasets used and/or analysed during the current study are available from the corresponding author and State Health Resource Centre, Chhattisgarh on reasonable request.

## Abbreviations

CHO: Community Health Officer
CHW: Community Health Worker
HLM: Health labour market
HLMA: Health labour market analysis
HRH: Human Resources for Health
HW: Health workers
HWF: Health workforce
LLMIC: Low- and low-to middle-income country
MBBS: Bachelor of Medicine and Bachelor of Surgery
PG: Post graduate
SHRC: State Health Resource Centre
SN: Staff nurse
UG: Under graduate
UHC: Universal health coverage
WHO: World Health Organization
