# Supplementary material for: Implementing a health labour market analysis to address health workforce gaps in a rural region of India
Source: Hum Resour Health. 2022 Jun 4;20:50. doi: 10.1186/s12960-022-00749-6 (PMC9167498; doi:10.1186/s12960-022-00749-6)
Supplement: Supplementary file 1 — Additional file 1: Table S1. List of stakeholders interviewed (qualitative). Table S2. List of quantitative data collected in Chhattisgarh. [file 12960_2022_749_MOESM1_ESM.docx]

**Additional File**

**Additional File Table S1: List of stakeholders interviewed (qualitative)**

| Stakeholder Category | Sub-category | No. of individuals interviewed during the stakeholder consultation to identify policy questions for HLMA | No. of individuals interviewed to identify issues underlying HRH gaps |
| --- | --- | --- | --- |
| State leadership of Department of Health | Department of Health and Family Welfare | 3 | 2 |
| State level officials directly involved in implementing HRH policies, regulation, recruitments | Directorate of Medical Education | 1 | 2 |
|  | Directorate of Health Services | 2 | 3 |
|  | Office of National Health Mission | 1 | 1 |
|  | State Medical Council | 1 | 1 |
|  | State Nursing Council | 1 | 2 |
| Teaching/training institutions | Medical college officials | 1 | 1 |
|  | Nursing college officials | 1 | 1 |
| Field officials | District government health officials | 1 | 2 |
|  | Sub-district officials | 1 | 2 |
| Health professionals | Specialists | 1 | 2 |
|  | UG doctors/students | 1 | 3 |
|  | Nurses | 1 | 2 |
|  | CHOs | 1 | 2 |
| Civil Society experts |  | 1 | 2 |
| Private sector representatives |  | 1 | 1 |

**Additional File Table S2: List of quantitative data collected in Chhattisgarh**

| **Sl.** | **Data** | **Period** | **Source** |
| --- | --- | --- | --- |
| 1 | No. of MBBS doctors registered in Chhattisgarh | 2000-2018 | Chhattisgarh Medical Council |
| 2 | No. of specialist faculty positions approved for government medical colleges in Chhattisgarh | 2016 to 2021 | Directorate of Medical Education, Chhattisgarh |
| 3 | No. of UG positions approved for government medical colleges and teaching hospitals in Chhattisgarh | 2016 to 2021 | Directorate of Medical Education, Chhattisgarh |
| 4 | No. of filled specialist faculty positions in government medical colleges in Chhattisgarh | 2016 to 2021 | Directorate of Medical Education, Chhattisgarh |
| 5 | No. of filled UG positions in government medical colleges and teaching hospitals in Chhattisgarh | 2016 to 2021 | Directorate of Medical Education, Chhattisgarh |
| 6 | No. of approved seats for UG students (MBBS) in government and private medical colleges in Chhattisgarh | 2000-2018 | Directorate of Medical Education, Chhattisgarh |
| 7 | No. of UG doctors produced by government and private medical colleges in Chhattisgarh | 2000-2018 | Directorate of Medical Education, Chhattisgarh |
| 8 | District-wise approved no. of permanent positions of UG doctors under Directorate of Health Services | 2016 to 2021 | Directorate Of Health Services, Chhattisgarh |
| 9 | District-wise approved no. of contractual positions of UG doctors in facilities of Directorate of Health Services under National Health Mission | 2016 to 2021 | Directorate Of Health Services, Chhattisgarh |
| 10 | Recruitment drives of UG doctors and no. of doctors recruited in each drive | 2000-2021 | Directorate Of Health Services, Chhattisgarh |
| 11 | Recruitment drives of specialist doctors and no. of doctors recruited in each drive | 2000-2021 | Directorate of Medical Education, Chhattisgarh |
| 12 | District-wise filled no. of permanent positions of UG doctors under Directorate of Health Services | 2016 to 2021 | Directorate Of Health Services, Chhattisgarh |
| 13 | District-wise filled no. of contractual positions of UG doctors in facilities of Directorate of Health Services under National Health Mission | 2016 to 2021 | Directorate Of Health Services, Chhattisgarh |
| 14 | District-wise no. of UG doctors working in facilities of Directorate of Health Services under the UG Bond scheme | 2016 to 2021 | Directorate Of Health Services, Chhattisgarh |
| 15 | No. of specialist doctors produced by government and private medical colleges in Chhattisgarh | 2000-2018 | Directorate of Medical Education, Chhattisgarh |
| 16 | District-wise approved no. of permanent positions of specialist doctors under Directorate of Health Services | 2016 to 2021 | Directorate Of Health Services, Chhattisgarh |
| 17 | District-wise approved no. of contractual positions of specialist doctors in facilities of Directorate of Health Services under National Health Mission | 2016 to 2021 | Directorate Of Health Services, Chhattisgarh |
| 18 | District-wise filled no. of permanent positions of specialist doctors under Directorate of Health Services | 2016 to 2021 | Directorate Of Health Services, Chhattisgarh |
| 19 | Age, gender and post-graduate specialty profile of specialist doctors working under Directorate of Health Services | 2018 | Directorate Of Health Services, Chhattisgarh |
| 20 | District-wise filled no. of contractual positions of specialist doctors in facilities of Directorate of Health Services under National Health Mission | 2016 to 2021 | Directorate Of Health Services, Chhattisgarh |
| 21 | District-wise no. of specialist doctors working in facilities of Directorate of Health Services under the Bond scheme | 2016 to 2021 | Directorate Of Health Services, Chhattisgarh |
| 22 | No. of post graduate doctors working in non-specialist positions under Directorate of Health Services | 2016 to 2021 | Directorate Of Health Services, Chhattisgarh |
| 23 | District-wise no. of registered private hospitals | 2018 | Directorate Of Health Services, Chhattisgarh |
| 24 | District-wise no. of registered private clinics | 2018 | Directorate Of Health Services, Chhattisgarh |
| 25 | No. of UG and specialist doctors working in private sector in Chhattisgarh | 2018 | Directorate Of Health Services, Chhattisgarh |
| 26 | No. of nurses registered in Chhattisgarh | 2000-2018 | Chhattisgarh Nursing Council |
| 27 | No. of approved seats for nursing in government and private nursing colleges in Chhattisgarh | 2000-2018 | Directorate of Medical Education, Chhattisgarh |
| 28 | No. of nurses produced by government and private nursing colleges in Chhattisgarh | 2000-2018 | Directorate of Medical Education, Chhattisgarh |
| 29 | No. of nurses working in private facilities | 2018 | Chhattisgarh Nursing Council |
| 30 | District-wise approved no. of permanent positions of nurses under Directorate of Health Services | 2016 to 2021 | Directorate Of Health Services, Chhattisgarh |
| 31 | District-wise approved no. of contractual positions of nurses in facilities of Directorate of Health Services under National Health Mission | 2016 to 2021 | Directorate Of Health Services, Chhattisgarh |
| 32 | District-wise filled no. of permanent positions of nurses under Directorate of Health Services | 2016 to 2021 | Directorate Of Health Services, Chhattisgarh |
| 33 | District-wise filled no. of contractual positions of nurses in facilities of Directorate of Health Services under National Health Mission | 2016 to 2021 | Directorate Of Health Services, Chhattisgarh |
| 34 | No. of approved seats of Community Health Officers (CHOs) in study centres | 2016 to 2021 | Office of National Health Mission, Chhattisgarh |
| 35 | No. of CHOs produced | 2016 to 2021 | Office of National Health Mission, Chhattisgarh |
| 36 | District-wise approved no. of positions of Community Health Officers (CHOs) | 2016 to 2021 | Office of National Health Mission, Chhattisgarh |
| 37 | District-wise filled no. of positions of Community Health Officers (CHOs) | 2016 to 2021 | Office of National Health Mission, Chhattisgarh |
